# Characterizing induced pluripotent stem cells and derived cardiomyocytes: insights from nano scale mass measurements and mechanical properties†

**DOI:** 10.1039/d3na00727h

**Published:** 2023-11-28

**Authors:** Irene C. Turnbull, Angelo Gaitas

**Affiliations:** a Cardiovascular Research Institute, Icahn School of Medicine at Mount Sinai New York NY 10029 USA irene.turnbull@mssm.edu; b The Estelle and Daniel Maggin Department of Neurology, Icahn School of Medicine at Mount Sinai New York NY 10029 USA angelo.gaitas@mssm.edu; c BioMedical Engineering & Imaging Institute, Leon and Norma Hess Center for Science and Medicine New York NY 10029 USA

## Abstract

Our study reveals that the nano-mechanical measures of elasticity and cell mass change significantly through induced pluripotent stem cell (iPSC) differentiation to cardiomyocytes, providing a reliable method to evaluate such processes. The findings support the importance of identifying these properties, and highlight the potential of AFM for comprehensive characterization of iPSC at the nanoscale.

## Abstract 2

Our study reveals that the nano-mechanical measures of elasticity and cell mass change significantly through induced pluripotent stem cell (iPSC) differentiation to cardiomyocytes, providing a reliable method to evaluate such processes.

## Graphical abstract

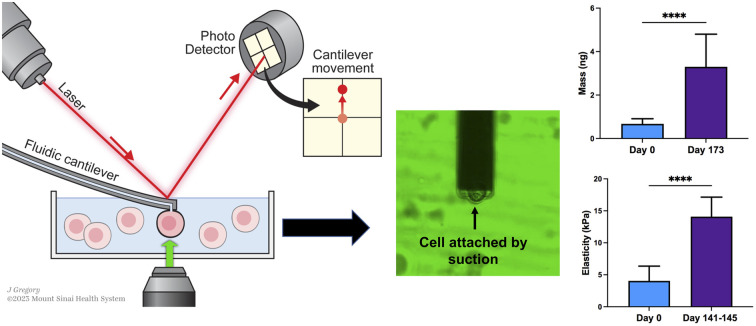

This research employs atomic force microscopy (AFM)^1–3^ to investigate single-cell characteristics of human induced pluripotent stem cells (iPSCs) and iPSCs-derived cardiomyocytes (iPSC-CMs) at the nanoscale. iPSCs, reprogrammed from mature cells like human skin cells, can be specialized into various types, including cardiomyocytes (CMs).^4^ The promise of iPSC-CMs extends to numerous areas such as cell therapy, drug testing, and investigating aspects of cardiac disease.^5–8^ They are increasingly recognized for their potential in drug evaluation, with particular interest for their use to predict subject-specific drug effects, thereby paving the way for personalized medicine.^9–15^ iPSC-CMs hold promise for heart tissue repair in regenerative medicine.^16^ Progress in these applications of hiPSC-CMs hinges on the ability to accurately characterize these cells at the single-cell level. Understanding their maturation status by adding new phenotypic markers is key.

Cell mass is an integral parameter which provides fundamental insights into cellular health, growth, and proliferative potential.^17^ Mass measurements at the single-cell level enable precise quantification of cellular growth rates, biomass production, and metabolic activities.^18–29^ Changes in cell mass are reflective of shifts in intracellular processes, including protein synthesis, cell division, and differentiation, thereby offering a valuable tool for monitoring these key cellular events.^19–29^ Therefore, measuring single-cell mass is crucial for characterizing cell populations, evaluating the impacts of external stimuli, and understanding cell cycle dynamics and disease processes at an unprecedented resolution. Similarly, single-cell elasticity is of significant importance as it is intrinsically linked to the cell's functionality and state of differentiation.^30^ The elasticity of a cell, or its stiffness, is a critical physical property that is affected by cytoskeletal organization and intracellular force generation mechanisms, often reflective of cellular behavior and biological processes such as proliferation, migration, and differentiation.^31^ Thus, understanding these mechanical properties at a single-cell level provides invaluable insights into cell physiology and pathology, helping to elucidate the underpinnings of cellular transformations and their role in tissue function and disease states.^19–29,32^

In this study, we employed two AFM techniques to evaluate the elasticity and mass of individual iPSCs and iPSC-CM.^32–37^ AFM has emerged as a key tool in biomechanics for measuring single-cell elasticity, allowing for nanoscale resolution in mapping the elastic properties of cells, thereby providing valuable insights into cell health, behavior, and response to various stimuli.^38–44^ Utilizing the innovative fluidic-AFM technique we measured single cell mass, the technique allows for the measurement of multiple individual cells using fluidic pressure to attached the live cells *in vitro*.^45–47^ We report for the first time that elasticity and mass measurements can discern cells pre- and post-differentiation. Thus, AFM can provide a detailed single-cell analysis of cell elasticity and cell mass to offer insights into iPSC differentiation.^48^

For cell source we used a well-validated healthy iPSC cell line (SKiPS-31.3).^49^ iPSCs were grown in StemFlex media on 6-well plates coated with hESC-qualified Matrigel in 5% CO_2_ incubator at 37 °C. iPSCs were differentiated into CMs by following a monolayer-based differentiation protocol with minor modifications.^50–52^ When iPSCs reached about 80% confluency, StemFlex media was replaced with basal medium (RPMI 1640 media plus 2% B27 supplement minus Insulin), and 10 μM CHIR99021 for 24 hours. After 24 hours, the media was replaced with basal medium without CHIR99021. Then, after 48 hours (day 3), the medium was replaced with basal medium plus 5 μM IWR-1. After 48 hours (day 5), the media was replaced with basal medium. On day 7, the medium was replaced with RPMI 1640 media plus 2% B27 supplement. Thereafter, the media was exchanged every 2 days. Starting on days 7–10 post-CHIR treatment, iPSC-CMs begin to display spontaneous contractions.

For elasticity measurements of the iPSCs on day 0, 70–80% confluent iPSCs were treated with ReLeSR (STEMCELL technologies; 1 ml per well of a 6-well plate), after 1 minute the ReLeSR was removed and the cells were maintained for 5–8 minutes in the incubator (5% CO_2_, 37 °C) while intermittently monitoring their detachment by visualization under the microscope. Then the cells were resuspended in Stemflex medium (1 ml per well of a 6-well plate) and the cells were plated onto a Matrigel coated glass-bottom cell culture dish. Stemflex medium was exchanged the following day. On day 3 after replating, the medium was exchanged and the cells were used for elasticity analysis. For the iPSC-CMs, the cells were maintained in culture for at least 141 days. Of note, during this time in culture replating was necessary when the cell monolayer acquired a mesh like morphology and were at risk of detaching from the plate. Five-seven days before the AFM elasticity measurements, the iPSC-CMs were treated with 0.25% trypsin. After 5–10 minutes the cells were resuspended in RPMI 1640 media plus 2% B27 supplement with 10% FBS (2 ml per 1 ml of trypsin). After centrifugation (300 g × 5 minutes) the supernatant was removed and the cells were resuspended in RPMI 1640 media plus 2% B27 supplement with 10% FBS and plated on Matrigel coated glass-bottom cell culture dish. After 24 hours, the media was replaced with RPMI 1640 media plus 2% B27 supplement, and every other day thereafter until AFM measurements were performed.

Elastic modulus of the cells was measured using a FlexAFM (Nanosurf AG, Switzerland), with inbuilt temperature controller and an inverted Axio Observer (Carl Zeiss) microscope. Nanosensors qp-SCONT-10 probes with a spring constant of ∼0.01 N m^−1^, and tip height of 8 μm were used for measurements (Fig. 1a and b). Elasticity measurements were performed on various iPSCs on day zero and on day 141–145 of differentiation. We measured multiple sites on each cell. Total number of sampled sites were: *n* = 150 for day zero and *n* = 104 for day 141–145. The elastic modulus, which was determined from the force curves through the application of the Hertz model, was computed using the Automated Nanomechanical Analysis (ANA) software provided by Nanosurf.

**Fig. 1:**
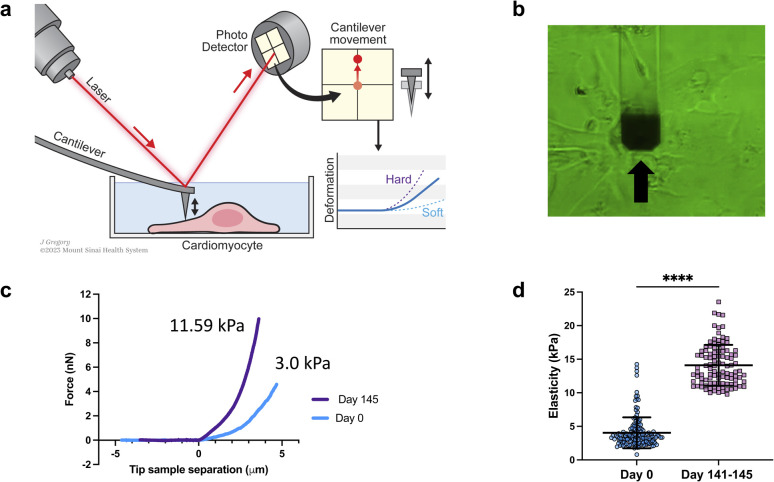
Elasticity analysis. (a) Schematic of elasticity measurements with AFM. Not to scale. (b) Microphotograph of AFM cantilever and iPSC-CMs for elasticity measurements. Of note, the cantilever is fabricated with SiO_2_ and therefore transparent to light, except on the distal portion which has a thin metal film for laser reflection (black arrow). (c) Representative raw force–distance curves, for cells on day 0 (blue line) and for day 145 (purple line). (d) Dot plot of the distribution of elasticity measurements at day 0 and at days 141–145 of differentiation. *N* = 150 for day-0 cells, and *N* = 104 for 141–145 day cells; bar and error bars represent the mean ± SD; ****P < 0.0001, unpaired *t*-test.

We conducted a comparison of iPSC-CM at two different time points: 0 days of differentiation and 141–145 days from differentiation. The results revealed that the older/differentiated cells exhibited an average modulus of elasticity of 14.1 ± 4 kPa (average ± standard deviation), whereas the cells at 0 days of differentiation had a modulus of 3 ± 2.3 kPa (Fig. 1c and d). During the differentiation and maturation process of iPSCs,^53^ the mechanical properties (stiffness/elasticity), undergo changes influenced by the organization and composition of the cytoskeleton, this includes enhanced alignment and organization of the cytoskeletal components—actin filaments, intermediate filaments, and microtubules.^53–55^ Immature cells have a less organized cytoskeleton and fewer myofibrils, resulting in lower stiffness. However, as cells mature, they develop more myofibrils, composed of actin and myosin proteins, which contribute to higher stiffness. The observed structural and functional alterations mirror those found in native cardiomyocyte development, which is characterized by the emergence of a more robust contractile apparatus and alterations in cellular stiffness. Our findings of increased stiffness following iPSC differentiation and maturation are consistent with prior reports from other research groups studying iPSCs.^53–55^ Notably, this study represents, to our knowledge, the first instance of a direct comparative analysis between iPSCs and their cardiomyocyte counterparts (iPSC-CMs).

For nano-gram mass measurements of iPSCs on day 0, when the iPSCs reached about 80% confluency, which is the point when the iPSC-CM differentiation starts (see above), the iPSCs were treated with ReLeSR; after 1 minute the ReLeSR was removed and the cells were maintained for 5–8 minutes in the incubator (5% CO_2_, 37 °C) while intermittently monitoring their detachment by visualization under the microscope. Then the cells were resuspended in Stemflex medium, with gentle pipetting for the cells to be singularized, and transferred to a microcentrifuge tube and prepared immediately for fluidic-AFM assessment. A glass bottom dish (without Matrigel coating) was filled with Stemflex culture medium, then a 100 μl aliquot from the cell suspension in the microcentrifuge tube was dispensed onto the center of the dish. The cells remained singularized and floating, rendering them accessible for capture by the microfluidic cantilever. For the iPSC-CMs, the cells were maintained in culture for 173 days; with replating as needed. On the day of the mass measurement, the iPSC-CMs were treated with 0.25% trypsin. After 5–10 minutes the cells were resuspended in RPMI 1640 media plus 2% B27 supplement with 10% FBS (2 ml per 1 ml of trypsin). After centrifugation (300 g × 5 minutes) the supernatant was removed and the cells were resuspended in RPMI 1640 media plus 2% B27 supplement with gentle pipetting while assuring that the cells were singularized, passed through a 100 μm cell strainer, and transferred to a microcentrifuge tube and prepared immediately for fluidic-AFM assessment. A glass bottom dish (without Matrigel coating) was filled with RPMI 1640 media plus 2% B27 supplement culture medium, then a 100 μl aliquot from the cell suspension of iPSC-CMs in the microcentrifuge tube was dispensed onto the center of the dish. The cells remained singularized and floating, rendering them accessible for capture by the microfluidic cantilever.

The experimental framework for measuring mass uses a microfluidic cantilever from Cytosurg AG, Switzerland. This cantilever includes a 4-micron aperture at the tip (Fig. 2a). The Sader method is used to determine the thermal resonance frequency. A pump provided by Cytosurge is connected to the cantilever, capable of exerting pressure from 800 mbar to 1000 mbar. The method, described in our previous work,^46^ involves moving the cantilever to position the tip of the fluidic probe above the cell of interest, then applying negative pressure to generate a suction force that attaches the cell to the opening of the fluidic probe. The measurement process involves recording the cantilever's resonance frequency while the cell is adhered to it; following cell detachment (achieved by applying positive pressure) the resonance frequency is subsequently re-measured in the absence of any attached cell (ESI videos 1 and 2†).

**Fig. 2:**
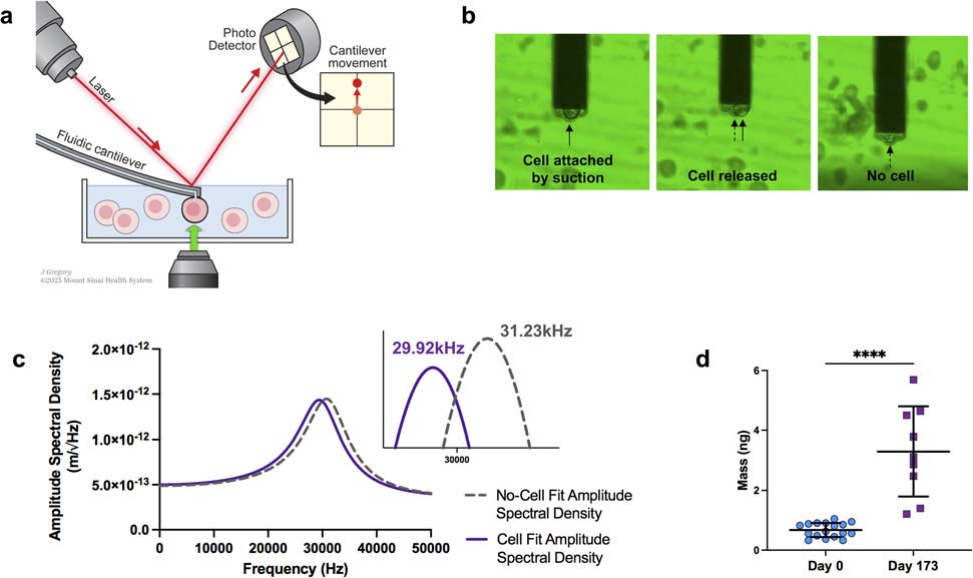
Mass analysis. (a) Schematic of mass measurements with fluidic-AFM, illustrating an iPSC-CM captured by the microfluidic cantilever. Not to scale. (b) Screen shots illustrate the sequence of events during the cell mass measurement process. The three static images show the cell attached (captured by the cantilever), the immediate moment that the cell is released and no longer attached to the cantilever, lastly the empty cantilever with no cell. Refer to ESI videos 1 and 2† to see the sequence in real-time. (c) Representative graph of the shift in resonance frequency post cell attachment (purple line) in comparison to the free/empty cantilever (gray dash line). Utilizing the frequencies and applying eqn (1), the calculated mass is approximately 5.69 ng. (d) Dot plot of cell mass measurements at day 0 and day 173 of differentiation. *N* = 17 for day 0, and *N* = 9 for day 173; bar and error bars represent the mean ± SD; ****P < 0.0001, unpaired *t*-test.

After cell detachment using positive pressure, the fluidic AFM cantilever is used to measure subsequent cells. This method allows the reuse of the same microfluidic cantilever for several measurements. An example of a microfluidic cantilever with a 4 μm aperture submerged in media with a captured iPSC-CM cell is shown in Fig. 2b. The entire measurement for each cell is completed in less than three minutes. A limitation in this application is the risk for clogging of the microfluidic probe, this can be minimized by careful selection according to cell size of the aperture tip and timing of negative pressures applied.

In our experiments, we measured cells at day 0, and at day 173 from the start of differentiation. We used an equation described in ref. 45, 46, 56 and 57 that allows for mass calculation based on the frequency peak changes (eqn (1)):1
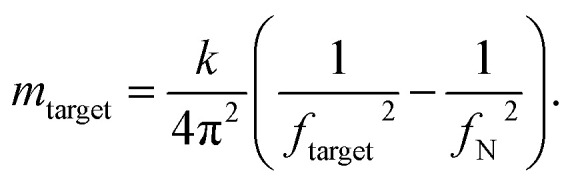
*m*_target_ is the measured mass of the target, *k* is the spring constant of the cantilever, and *f*_N_ and *f*_target_ are the resonance frequencies in liquid without and with cell attachment (Fig. 2c). The average mass of cells at day 0 was 0.67 ± 0.24 ng. For cells at 173 days from start of differentiation, the average mass was 3.30 ng ± 1.50 ng (Fig. 2d). The data clearly shows a significant difference in cell mass before and after differentiation.

While we anticipate that the general trend of increased stiffness and mass upon differentiation will hold true across various cell types, the absolute values and variability might differ. In a heterogeneous cell population, a larger variability in mechanical properties and cell mass is expected. The strength of our method lies in its ability to perform single-cell analyses; even in heterogeneous population, the mechanical properties and mass can be individually assessed.

In conclusion, for the tested iPSC cell line, we found that mass measurements and mechanical properties can serve as critical morphological markers for monitoring the differentiation of iPSC to iPSC-CMs. Our investigation showed a notable difference in cell mass and elasticity values pre- and post-differentiation, demonstrating our ability to distinguish between these stages in this specific cell line. While our findings are promising, further studies are required to determine if this ability extends universally to other cell lines and types. Our findings underscore the potential of AFM as a comprehensive tool for single-cell characterization at the nanoscale level. Both techniques can be used for a more detailed monitoring of differentiation and maturation of iPSC.^33–35^

The integrative and non-invasive nature of mass measurements offers great promise especially when combined with other AFM techniques. Fluidic AFM probes, for instance, not only perform mass measurements, but also can be used to extract genomic content of individual cells, thereby providing insights into the nuances of iPSC differentiation enhancing our understanding of iPSC differentiation processes.^58^ However, it is important to note that the eqn (1) employed in our study serves as an approximation. Its application, especially in a liquid medium, is not entirely accurate because it assumes there is no damping and operates under the assumption of *a* point mass.^59^ Improvements can be achieved by using more accurate mathematical modelling to derive mass and employing different cantilever materials and designs.^60^ Incorporating active vibration strategies, instead of the thermal tune method,^61,62^ can increase the amplitude of oscillation and in turn reduce the minimum detectable mass. Techniques such as photo-thermal modulation (where thermally induced stress is applied with a laser near the cantilever's fixed end),^63,64^ piezoelectric actuation,^65–67^ thermomechanical actuation,^68–70^ and magnetic excitation^71–73^ are potential avenues that can be explored to further improve the resolution of these measurements in liquids.

## Conflicts of interest

There are no conflicts to declare.

## Supplementary Material

NA-006-D3NA00727H-s001

NA-006-D3NA00727H-s002

NA-006-D3NA00727H-s003
