# Supplementary material for: Characterizing induced pluripotent stem cells and derived cardiomyocytes: insights from nano scale mass measurements and mechanical properties
Source: Nanoscale Adv. 2023 Nov 28;6(4):1059–64. doi: 10.1039/d3na00727h (PMC10863719; doi:10.1039/d3na00727h)
Supplement: NA-006-D3NA00727H-s001 [file NA-006-D3NA00727H-s001.pdf]

Electronic Supplementary Material (ESI) for Nanoscale Advances.
This journal is © The Royal Society of Chemistry 2023

Supplemental Video 1. Cell attachment for Mass measurement. Video shows a single cell being captured by the cantilever using suction. After capture, the cantilever is moved up and away from that area, the cell remains attached for measurement. In the video, there is a loss of focus on the cantilever when it is moved up because the focal plane remained at its original position. The cantilever is positioned as distant from the surface as feasible to mitigate any potential interference during its vibration for resonance frequency change measurement.

Supplemental Video 2. Cell release. Video shows the cell is being released from the cantilever using positive pressure. Following its release, the cantilever is moved up and re-enters the focal plane, ensuring the complete detachment of the cell from the cantilever.
